# Post COVID‐19 condition diagnosis: A population‐based cohort study of occurrence, associated factors, and healthcare use by severity of acute infection

**DOI:** 10.1111/joim.13584

**Published:** 2022-12-07

**Authors:** Pontus Hedberg, Fredrik Granath, Judith Bruchfeld, Johan Askling, Daniel Sjöholm, Michael Fored, Anna Färnert, Pontus Naucler

**Affiliations:** ^1^ Division of Infectious Diseases Department of Medicine Solna Karolinska Institutet Stockholm Sweden; ^2^ Department of Infectious Diseases Karolinska University Hospital Stockholm Sweden; ^3^ Clinical Epidemiology Division Department of Medicine Solna Karolinska Institutet Stockholm Sweden; ^4^ Rheumatology Theme Inflammation and Ageing Karolinska University Hospital Stockholm Sweden

**Keywords:** COVID‐19, long‐term outcomes, post COVID‐19 condition, SARS‐CoV‐2, sequelae

## Abstract

**Background:**

The occurrence and healthcare use trajectory of post COVID‐19 condition (PCC) is poorly understood. Our aim was to investigate these aspects in SARS‐CoV‐2‐positive individuals with and without a PCC diagnosis.

**Methods:**

We conducted a population‐based cohort study of adults in Stockholm, Sweden, with a verified infection from 1 March 2020 to 31 July 2021, stratified by the severity of the acute infection. The outcome was a PCC diagnosis registered any time 90–360 days after a positive test. We performed Cox regression models to assess baseline characteristics associated with the PCC diagnosis. Individuals diagnosed with PCC were then propensity‐score matched to individuals without a diagnosis to assess healthcare use beyond the acute infection.

**Results:**

Among 204,805 SARS‐CoV‐2‐positive individuals, the proportion receiving a PCC diagnosis was 1% among individuals not hospitalized for their COVID‐19 infection, 6% among hospitalized, and 32% among intensive care unit (ICU)–treated individuals. The most common new‐onset symptom diagnosis codes among individuals with a PCC diagnosis were fatigue (29%) among nonhospitalized and dyspnea among both hospitalized (25%) and ICU‐treated (41%) individuals. Female sex was associated with a PCC diagnosis among nonhospitalized and hospitalized individuals, with interactions between age and sex. Previous mental health disorders and asthma were associated with a PCC diagnosis among nonhospitalized and hospitalized individuals. Among individuals with a PCC diagnosis, the monthly proportion with outpatient care was substantially elevated up to 1 year after acute infection compared to before, with substantial proportions of this care attributed to PCC‐related care.

**Conclusion:**

The differential association of age, sex, comorbidities, and healthcare use with the severity of the acute infection indicates different trajectories and phenotypes of PCC, with incomplete resolution 1 year after infection.

## Graphical abstract

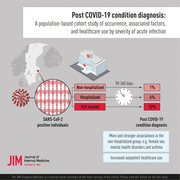

## Background

Long‐term effects following COVID‐19 have been described from multiple organ systems [1, 2]. The terminology to describe these varies, including “post‐acute COVID‐19 syndrome,” “post‐acute sequelae of COVID‐19” (PASC), “long COVID”, and post COVID‐19 condition (PCC) [3]. The occurrence and determinants of these long‐term effects are poorly characterized, with estimates ranging from below 10% to above 50% [4]. Such varying proportions may come from differences between study populations in the severity of the initial infection, but also from different case definitions and assessment periods, emphasizing the need for more harmonized definitions of PCC.

In September 2020, the World Health Organization (WHO) issued an International Classification of Disease 10th revision (ICD‐10) code for PCC (U09.9), and in October 2021, a WHO Delphi consensus definition of PCC was released [5, 6]. According to the definition, “Post COVID‐19 condition occurs in individuals with a history of probable or confirmed SARS‐CoV‐2 infection, usually three months from the onset of COVID‐19 with symptoms that last for at least two months and cannot be explained by an alternative diagnosis.” The many symptoms implicated in PCC include fatigue and malaise, dyspnea, and cognitive symptoms [2, 5].

Knowledge on the occurrence of PCC and its associated characteristics and effects on healthcare utilization is sparse, with a limited understanding of how the pre‐existing health status possibly modifies the risk across different severities of SARS‐CoV‐2 infection [7]. Accordingly, the aim of this study was to investigate: (i) the occurrence of a PCC diagnosis, (ii) sociodemographic and health status factors associated with PCC diagnosis, and (iii) healthcare use before and after infection in individuals with and without a PCC diagnosis.

## Methods

### Study design and population

We conducted a register‐ and population‐based cohort study of PCC diagnosis among residents within Stockholm County (approximate population 2.4 million) with a verified SARS‐CoV‐2 infection. Individuals 18 years of age and above with a polymerase chain reaction (PCR) positive SARS‐CoV‐2 test from 1 March 2020 to 31 July 2021 were included to allow for at least 6 months of follow‐up, which ended on 15 February 2022. To assure adequate capture of baseline characteristics, the study population was restricted to individuals residing in Stockholm County from 3 years before to 90 days after the first positive SARS‐CoV‐2 test. Individuals dying before 90 days after their first positive PCR test, or with less than 90 days of follow‐up after hospital discharge, were excluded. The need for consent was waived by the Swedish Ethical Review Authority (Dnr 2018/1030‐31, COVID‐19 research amendment Dnr 2020‐01385) since analyses are based on retrospectively collected data from the administrative health registry.

### Data sources and linkages

In Sweden, each resident has a personal identification code, enabling population‐based studies with complete follow‐up using health‐record linkages [8]. Data were linked from: (i) the Stockholm regional healthcare data warehouse (VAL), including demographics, medical diagnoses, inpatient stays, outpatient specialist visits and primary care visits reimbursed by Region Stockholm (covers 94% of all visits in primary care, *personal communication Göran Lord, Region Stockholm*), and drug prescriptions [9, 10]; (ii) the Swedish Intensive Care Registry (SIR), including patients admitted to an intensive care unit (ICU) [11]; (iii) SmiNet, containing all PCR SARS‐CoV‐2‐positive test results, which were notified in accordance with the Communicable Diseases Act [12]; and (iv) Statistics Sweden, containing socioeconomic information from before the COVID‐19 epidemic [13].

### Study outcome and follow‐up

The study outcome was a PCC diagnosis (ICD‐10 code U09.9) in primary care, outpatient specialist care, or inpatient care. We included U09.9 diagnoses registered at least 90 days after a SARS‐CoV‐2‐positive test in accordance with the case definition from the WHO [5]. The PCC diagnosis code was introduced in Sweden in October 2020, and national guidelines state that the diagnosis should not be given during acute infection [14]. Study subjects were followed for the outcome from 90 to 360 days after the first positive SARS‐CoV‐2 test. Subjects were censored at 360 days, 15 February 2022, the date of death, or the date of moving out of Stockholm County, whichever occurred first.

### Variable definitions

Descriptions of the study variables are available in Table S1. The severity of SARS‐CoV‐2 infection was categorized as not hospitalized with COVID‐19 (nonhospitalized), hospitalized with COVID‐19 without treatment in the ICU (hospitalized), and hospitalized with COVID‐19 with treatment in the ICU (ICU treated). We assessed symptom‐based diagnosis codes before and after the acute infection, corresponding to 19 of 25 symptoms included in the WHO PCC case definition (Table S1). Blurred vision, cognitive dysfunction, memory issues, menstrual problems, allergies, and postexertional malaise were excluded due to a lack of specific information in the data sources used. Sociodemographic data collected included the region of birth, residential area deprivation, and the number of days with sickness benefit during 2019. Comorbidity data were based on conditions with an increased risk of severe COVID‐19 [15]. Primary care, outpatient specialist care, and inpatient care were assessed from 300–30 days before and 90–360 days after a positive SARS‐CoV‐2 test

### Statistical methods

All analyses were performed stratified by the severity of SARS‐CoV‐2 infection (nonhospitalized, hospitalized, ICU treated) to account for differential follow‐up strategies within the healthcare system, hence influencing the likelihood of receiving a post‐COVID‐19 diagnosis.

The occurrence of PCC was described as the proportion with a PCC diagnosis registered any time from 90 to 360 days after the first positive SARS‐CoV‐2 test. Baseline characteristics of individuals with and without PCC were presented using descriptive statistics with continuous variables as medians with interquartile ranges and categorical variables as numbers with percentages. The proportion of individuals with PCC having symptom diagnoses consistent with those included in the case definition of PCC was analyzed. These analyses were performed only for individuals with a PCC diagnosis because the diagnosis guidelines state that symptom diagnoses should be registered in conjunction with the PCC diagnosis, possibly leading to differences in recording procedures between individuals with and without PCC [14].

Fixed‐effects Cox proportional hazards regression models were used to explore factors associated with PCC. Hazard ratios were presented with corresponding 95% confidence intervals (CIs). All models were stratified on the month of first positive test to account for similar follow‐up time and probability of receiving a PCC diagnosis. In the first model, we included as independent variables age, sex, and the interaction between age and sex. Among hospitalized and ICU‐treated individuals, we also fitted separate models including the length of hospitalization/ICU stay. We then investigated the association between comorbidities, previous healthcare use, days with sickness benefit during 2019, region of birth, and residential area and P diagnosis. These models included age, sex, the interaction between age and sex, and each investigated factor. For all the above‐described models, age was included as a continuous variable, using restricted cubic splines with four knots [16]. Three variables—residential area, region of birth, and days with sickness benefit—had low missing data (<0.5% for all variables), for which only complete case analyses were performed.

To investigate healthcare use after the acute infection, individuals with a PCC diagnosis were matched to up to three individuals (for nonhospitalized and hospitalized individuals) and to one individual (for ICU‐treated individuals) without a PCC diagnosis. Exact matching was used for the month of first positive test, sex, age group, and nearest neighbour propensity score matching for all studied comorbidities individually and the total number of comorbidities, region of birth, residential area type, and number of days with sickness benefit during 2019, as well as the number of previous primary care, outpatient specialist care, and inpatient care visits. Individuals with missing data were excluded from the matching procedure (<0.5% of the study population). Covariate balance before and after matching was assessed using absolute standardized mean differences (SMDs).

Healthcare use before and after the acute infection was analyzed descriptively using the crude proportions with at least one healthcare visit per month in relation to the first positive SARS‐CoV‐2 test. For each month, only individuals with full follow‐up that specific month were included, and for individuals with a PCC diagnosis, the first visit where this diagnosis was given was excluded. For overall primary care, outpatient specialist care, and inpatient care visit rates, we used difference‐in‐differences analyses to assess healthcare use after, compared to before, the acute infection in individuals with PCC and matched controls [17]. Difference‐in‐differences analyses constitute a quasi‐experimental approach well established in public health research, as has been reviewed and explained in more detail [18].

We estimated 95% CIs based on robust standard errors. All analyses were conducted with R version 4.1.0.

## Results

Among 230,189 SARS‐CoV‐2‐positive individuals, 204,805 (89%) were included in the study population. Reasons for exclusion were death before the start of follow‐up (90 days after the SARS‐CoV‐2‐positive test) (*n* = 4889), moving to or from the region from 3 years before the SARS‐CoV‐2‐positive test to the start of follow‐up (*n* = 20,428), and having less than 3 months of follow‐up after hospital discharge (*n* = 67). Of the 204,805 individuals, 191,459 were nonhospitalized in connection with their positive SARS‐CoV‐2 test, 12,070 were hospitalized without ICU treatment, and 1276 were ICU treated. In total, 96% (*n* = 196,012) and 59% (*n* = 120,384) were followed up until 270 and 360 days after their positive test, respectively. Baseline characteristics—stratified on disease severity and occurrence of post‐COVID‐19 diagnosis during follow‐up—are presented in Table 1.

**Table 1:** Characteristics of the study population stratified by the severity of acute infection and post‐COVID‐19 diagnosis

	Nonhospitalized (*n* = 191,459)		Hospitalized (*n* = 12,070)		ICU treated (*n* = 1276)	
Variable	No post COVID‐19 (*n* = 189,496)	Post COVID‐19 (*n* = 1963)	No post COVID‐19 (*n* = 11,377)	Post COVID‐19 (*n* = 693)	No post COVID‐19 (*n* = 866)	Post COVID‐19 (*n* = 410)
Female sex	99,961 (52.8)	1399 (71.3)	4876 (42.9)	322 (46.5)	250 (28.9)	137 (33.4)
Age, years	42.0 (31.0, 53.0)	48.0 (40.0, 56.0)	65.0 (52.0, 78.0)	60.0 (52.0, 70.0)	62.0 (53.0, 69.0)	59.0 (51.0, 66.0)
18–29	38,667 (20.4)	119 (6.1)	246 (2.2)	6 (0.9)	19 (2.2)	7 (1.7)
30–39	43,185 (22.8)	354 (18.0)	681 (6.0)	25 (3.6)	48 (5.5)	21 (5.1)
40–49	45,200 (23.9)	591 (30.1)	1368 (12.0)	103 (14.9)	96 (11.1)	62 (15.1)
50–59	36,317 (19.2)	561 (28.6)	2208 (19.4)	192 (27.7)	215 (24.8)	119 (29.0)
60–69	16,570 (8.7)	257 (13.1)	2154 (18.9)	189 (27.3)	274 (31.6)	125 (30.5)
70–79	5821 (3.1)	64 (3.3)	2251 (19.8)	116 (16.7)	180 (20.8)	71 (17.3)
>80	3736 (2.0)	17 (0.9)	2469 (21.7)	62 (8.9)	34 (3.9)	5 (1.2)
Comorbidities						
Asthma	10,085 (5.3)	230 (11.7)	1163 (10.2)	119 (17.2)	73 (8.4)	54 (13.2)
Cancer	5107 (2.7)	56 (2.9)	1334 (11.7)	71 (10.2)	66 (7.6)	31 (7.6)
Cerebrovascular disease	1694 (0.9)	22 (1.1)	825 (7.3)	26 (3.8)	33 (3.8)	13 (3.2)
Chronic kidney disease	1456 (0.8)	14 (0.7)	866 (7.6)	39 (5.6)	42 (4.8)	14 (3.4)
Chronic liver disease	637 (0.3)	13 (0.7)	148 (1.3)	10 (1.4)	15 (1.7)	6 (1.5)
Chronic lung disease	1933 (1.0)	33 (1.7)	1113 (9.8)	54 (7.8)	48 (5.5)	20 (4.9)
Diabetes (type 1 or type 2)	7236 (3.8)	92 (4.7)	2453 (21.6)	131 (18.9)	216 (24.9)	98 (23.9)
Heart disease	7586 (4.0)	95 (4.8)	2765 (24.3)	110 (15.9)	135 (15.6)	62 (15.1)
Hypertension	20,087 (10.6)	298 (15.2)	5175 (45.5)	277 (40.0)	400 (46.2)	164 (40.0)
Immunocompromised state	4427 (2.3)	84 (4.3)	1009 (8.9)	59 (8.5)	57 (6.6)	38 (9.3)
Mental health disorder	36,579 (19.3)	720 (36.7)	2322 (20.4)	195 (28.1)	154 (17.8)	89 (21.7)
Neurological disease	3921 (2.1)	36 (1.8)	1098 (9.7)	42 (6.1)	36 (4.2)	15 (3.7)
Number of comorbidities						
0	120,723 (63.7)	875 (44.6)	3193 (28.1)	191 (27.6)	263 (30.4)	126 (30.7)
1	48,015 (25.3)	696 (35.5)	2578 (22.7)	176 (25.4)	229 (26.4)	109 (26.6)
2	13,462 (7.1)	258 (13.1)	2144 (18.8)	155 (22.4)	199 (23.0)	80 (19.5)
>2	7296 (3.9)	134 (6.8)	3462 (30.4)	171 (24.7)	175 (20.2)	95 (23.2)
Previous healthcare use						
Primary care						
0 visits	101,793 (53.7)	624 (31.8)	3499 (30.8)	204 (29.4)	325 (37.5)	145 (35.4)
1–2 visits	62,045 (32.7)	693 (35.3)	4233 (37.2)	258 (37.2)	308 (35.6)	154 (37.6)
3–4 visits	16,505 (8.7)	328 (16.7)	1973 (17.3)	111 (16.0)	135 (15.6)	64 (15.6)
>4 visits	9153 (4.8)	318 (16.2)	1672 (14.7)	120 (17.3)	98 (11.3)	47 (11.5)
Outpatient specialist care						
0 visits	114,401 (60.4)	880 (44.8)	4366 (38.4)	249 (35.9)	375 (43.3)	175 (42.7)
1–2 visits	44,909 (23.7)	555 (28.3)	3096 (27.2)	215 (31.0)	233 (26.9)	109 (26.6)
3–4 visits	15,091 (8.0)	237 (12.1)	1579 (13.9)	85 (12.3)	105 (12.1)	46 (11.2)
>4 visits	15,095 (8.0)	291 (14.8)	2336 (20.5)	144 (20.8)	153 (17.7)	80 (19.5)
Any inpatient visit	9364 (4.9)	94 (4.8)	2094 (18.4)	80 (11.5)	115 (13.3)	39 (9.5)
Sociodemographic variables						
Days with sickness benefit during 2019						
0	172,340 (91.3)	1563 (79.6)	10,434 (91.8)	566 (81.7)	788 (91.0)	349 (85.1)
1–30	7410 (3.9)	178 (9.1)	336 (3.0)	55 (7.9)	25 (2.9)	14 (3.4)
>30	9014 (4.8)	222 (11.3)	600 (5.3)	72 (10.4)	53 (6.1)	47 (11.5)
Region of birth						
Africa	4919 (2.6)	37 (1.9)	544 (4.8)	31 (4.5)	71 (8.2)	26 (6.3)
America	5213 (2.8)	75 (3.8)	472 (4.2)	34 (4.9)	48 (5.5)	28 (6.8)
Asia/Oceania	24,389 (12.9)	270 (13.8)	2598 (22.8)	121 (17.5)	197 (22.7)	94 (22.9)
Europe	16,092 (8.5)	202 (10.3)	1465 (12.9)	97 (14.0)	127 (14.7)	59 (14.4)
Sweden	138,211 (73.2)	1379 (70.2)	6294 (55.3)	410 (59.2)	423 (48.8)	203 (49.5)
Residential area type						
1 (most deprived)	4812 (2.6)	28 (1.4)	733 (6.5)	22 (3.2)	64 (7.4)	16 (3.9)
2	12,889 (6.8)	126 (6.4)	1350 (11.9)	79 (11.4)	115 (13.3)	63 (15.4)
3	24,853 (13.2)	252 (12.8)	1969 (17.3)	112 (16.2)	152 (17.6)	76 (18.6)
4	105,387 (55.8)	1138 (58.0)	5672 (49.9)	342 (49.6)	442 (51.2)	188 (46.1)
5 (least deprived)	40,758 (21.6)	419 (21.3)	1635 (14.4)	135 (19.6)	90 (10.4)	65 (15.9)

*Note*: Numeric values are presented as median (interquartile range) and categorical values are presented as number (percentage).

Abbreviations: COVID‐19, coronavirus disease 2019; ICU, intensive care unit.

### Occurrence of post COVID‐19 condition diagnosis

**Fig. 1:**
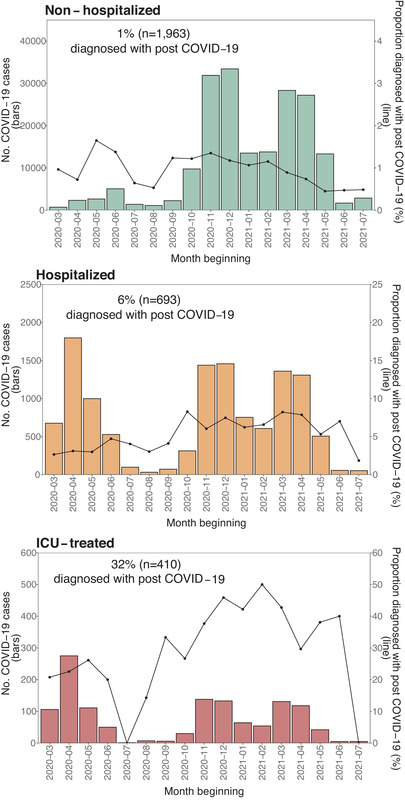
Monthly number of COVID‐19 cases (bars) and proportion subsequently diagnosed with post COVID‐19 condition (line) by the severity of acute infection. Note: The overall proportion diagnosed with a post COVID‐19 condition is presented as text in the upper part of each graph. Abbreviations: COVID‐19, coronavirus disease 2019; ICU, intensive care unit.

Figure 1 displays the number of individuals with a positive test and the proportion of those who received a PCC diagnosis. The proportion receiving a PCC diagnosis during follow‐up was 1.5% (*n* = 3066), being 1% (*n* = 1963) among nonhospitalized, 6% (*n* = 693) among the hospitalized, and 32% (*n* = 410) among the ICU‐treated individuals. Among hospitalized and ICU‐treated individuals, the occurrence of PCC diagnosis was associated with an increased length of stay in the hospital/ICU during the acute infection (Fig. S1). Among nonhospitalized individuals, 83% had their PCC diagnosed in primary care only, compared to 72% and 31% for the hospitalized and ICU‐treated individuals, respectively.

The most common new‐onset symptom diagnosis codes among nonhospitalized individuals with PCC diagnosis were fatigue (29%) and anxiety (21%) (Fig. S2). Among hospitalized or ICU‐treated individuals, these were dyspnea (25% hospitalized, 41% ICU) and fatigue (22% hospitalized, 29% ICU). Before the acute SARS‐CoV‐2 infection, 53% of nonhospitalized, 48% of hospitalized, and 41% of ICU‐treated individuals had one or more symptom diagnoses included in the WHO PCC case definition recorded. The most common symptom diagnosis registered before the infection was anxiety for the nonhospitalized (16%) and hospitalized (12%) and muscle pain (12%) for the ICU treated.

The proportion with a suspected SARS‐CoV‐2 re‐infection (two positive tests at least 90 days apart) during follow‐up was 5% (*n* = 10,266), being 5% (*n* = 9924) among nonhospitalized, 3% (*n* = 322) among hospitalized, and 2% (*n* = 20) among ICU‐treated individuals.

### Sociodemographic and health status factors associated with a post COVID‐19 condition diagnosis

**Fig. 2:**
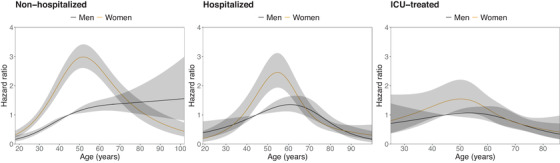
Associations between age, sex, and post COVID‐19 condition by the severity of acute infection. Note: The model included age, sex, and the interaction between age and sex and these were stratified on the calendar month of the first positive test. Age was included as a continuous variable, using restricted cubic splines with four knots. Abbreviation: ICU, intensive care unit.

We first investigated the association between sex, age, and PCC (Fig. 2). Among ICU‐treated patients, no clear‐cut associations were observed, while among nonhospitalized and hospitalized individuals, the occurrence of PCC was most common among middle‐aged individuals, which for both groups was more pronounced among females.

**Fig. 3:**
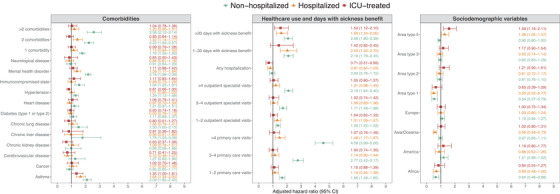
Associations between comorbidities, healthcare use, days with sickness benefit, and sociodemographic variables with post COVID‐19 condition diagnosis. Note: Cox proportional hazards regression models stratified on the month of first positive SARS‐CoV‐2 test, adjusted for age (restricted cubic splines with four knots), sex, and the interaction between age and sex were used for each factor separately. Absence of each comorbidity, zero comorbidities, zero days with sickness benefit, zero primary care visits, zero outpatient specialist care visits, zero hospitalizations, area type 4, and being born in Sweden were used as references. Residential area type 1 represents the most deprived areas and type 5 represents the least deprived areas; see the supplement for details. Abbreviation: ICU, intensive care unit.

Figure 3 shows the associations for comorbidities, previous healthcare use, days with sickness benefit, region of birth, and residential area with PCC diagnosis. The strongest associations for comorbidities were observed among nonhospitalized individuals, particularly mental health disorder (PCC: 37%, no PCC: 19%, adjusted hazard ratio [aHR] 2.18 [95% CI 1.98–2.39]) and asthma (PCC: 12%, no PCC: 5%, aHR 2.09 [95% CI 1.82–2.40]). Mental health disorders and asthma were also positively associated with PCC diagnosis among hospitalized individuals, whereas for ICU‐treated individuals, none of the individual comorbidity categories showed a clear association with PCC diagnosis.

For all three severity groups, more than 30 days with sickness benefit during 2019 was associated with PCC diagnosis. Four previous primary care visits were strongly associated with PCC diagnosis among nonhospitalized individuals (PCC: 16%, no PCC: 5%, aHR 4.58 [95% CI 3.99–5.26]). There were no clear observations for hospitalized and ICU‐treated individuals with regard to previous outpatient healthcare use. The ICD‐10 diagnosis codes registered during the previous outpatient and inpatient care visits are summarized for individuals with and without PCC diagnosis in Figs S3 and S4. Among individuals with PCC diagnosis, the two most common previously registered diagnoses were neurotic and stress‐related disorders (13%) and hypertension (9%) among nonhospitalized, hypertension (25%) and eye disease (16%) among hospitalized, and hypertension (25%) and type 2 diabetes (13%) among ICU‐treated individuals.

Among nonhospitalized individuals, being born in Africa was associated with a decreased risk of PCC diagnosis (aHR 0.64 [95% CI 0.46–0.88]), whereas for hospitalized individuals, being born in Asia/Oceania was associated with a decreased risk of PCC diagnosis (aHR 0.59 [95% CI 0.48–0.73]). For nonhospitalized and hospitalized individuals, living in a socioeconomically deprived area was associated with a decreased risk of PCC diagnosis.

### Healthcare usage after the acute infection

We analyzed primary care, outpatient specialist care, and inpatient care before and after the infection in individuals with a PCC diagnosis and matched controls. Descriptive statistics of the matched cohorts are presented in Table S2. Good covariate balance was achieved for nonhospitalized and hospitalized individuals, whereas for ICU‐treated individuals, four out of 19 variables had SMD >0.1 after matching (Figs S5–S7).

**Fig. 4:**
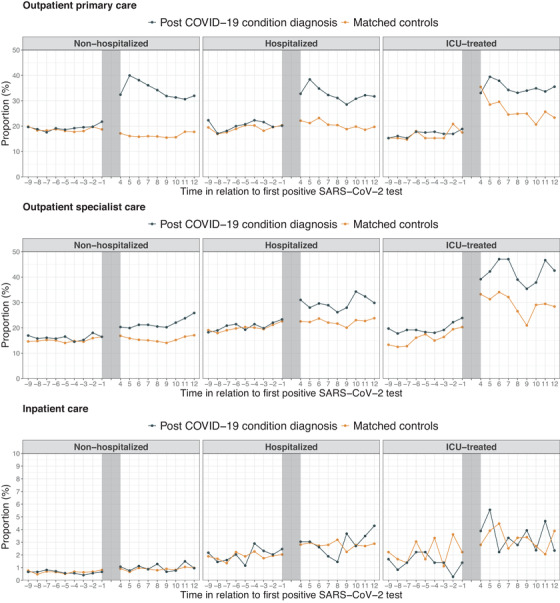
Primary care, outpatient specialist care, and inpatient care use from 9 months before to 12 months after SARS‐CoV‐2 infection in individuals with post COVID‐19 condition diagnosis and matched controls. Note: The upper panels show the proportion with a primary care medical doctor visit per month. The middle panels show the proportion with an outpatient specialist care medical doctor visit per month. The lower panels show the proportion with an inpatient visit per month. For each month after the infection, only individuals with full follow‐up for each month were included per month. The visits where individuals with post COVID‐19 condition diagnosis were first diagnosed with post COVID‐19 condition were excluded from the analyses. Abbreviations: COVID‐19, coronavirus disease 2019; ICU, intensive care unit; SARS‐CoV‐2, severe acute respiratory syndrome coronavirus 2.

The proportion with at least one primary care, outpatient specialist care, or inpatient care visit per month from 9 months before to 12 months after the first positive SARS‐CoV‐2 test is presented in Fig. 4. Among nonhospitalized individuals with a PCC diagnosis, a substantial increase in the proportion with monthly primary care visits after (vs. before) the infection was observed, persisting 12 months after the first positive SARS‐CoV‐2 test. For the matched population controls, no increase in the proportion with a primary care visit was observed. Similar trends were observed for hospitalized individuals, whereas for ICU‐treated individuals, the proportion with primary care visits after (vs. before) the acute infection was increased for both individuals with a PCC diagnosis and for their matched controls. Among individuals with a PCC diagnosis, the monthly proportion of a primary care visit ranged from 30% to 40% during months 4–12, resulting in a monthly incidence rate of primary care visits of 0.33 visits for all three severities of the acute infection.

With regard to outpatient specialist care, the clearest increase was observed after (vs. before) infection in ICU‐treated individuals. No clear trends were observed for inpatient care use after (vs. before) the acute infection in the three severity groups.

We then assessed the monthly primary care, outpatient specialist care, and inpatient care visit rates before and after the infection using difference‐in‐differences analyses. For all three COVID‐19 severity groups, the monthly primary care visit rate was significantly increased in individuals with a PCC diagnosis compared to matched controls (nonhospitalized: 0.23 [95% CI 0.21–0.25] visits, hospitalized: 0.14 [95% CI 0.10–0.18] visits, ICU treated: 0.10 [95% CI 0.03–0.16] visits). For outpatient specialist care, the monthly visit rate after compared to before infection was significantly increased among nonhospitalized individuals (0.09 [95% CI 0.06–0.13] visits), but not for hospitalized (0.09 [95% CI −0.01 to 0.19] visits) and ICU‐treated individuals (0.18 [95% CI −0.02 to 0.38] visits). For inpatient care, no significant differences were observed. Around 70%–80% of all primary care visits among nonhospitalized individuals with a PCC diagnosis had a PCC‐related diagnosis registered, compared to around 50%–70% for hospitalized individuals and 50% for ICU‐treated individuals (Fig. S8). These proportions in outpatient specialist care were lower for all three severity groups, ranging from around 20% to 40%.

## Discussion

In this population‐based cohort study of 204,805 SARS‐CoV‐2‐positive individuals, we found the occurrence of PCC diagnosis to differ substantially depending on the severity of the acute infection. Female sex was associated with a PCC diagnosis among nonhospitalized and hospitalized individuals, being the most pronounced among middle‐aged individuals. Comorbidities, particularly mental health disorder and asthma, and previous healthcare use had stronger associations with PCC diagnosis among nonhospitalized individuals compared to hospitalized and ICU‐treated individuals. Substantial increases in outpatient healthcare usage after compared to before the acute infection were observed, persisting up to 12 months after infection, with a substantial proportion of this care attributed to PCC.

Previous reports on effects beyond the SARS‐CoV‐2 infection are primarily based on individuals hospitalized during the acute infection. In a systematic review, encompassing 39 studies on long COVID, only four studies followed up nonhospitalized individuals [2]. Our findings of major differences in occurrence of PCC diagnosis by different severities of the acute infection are in line with a report of 181,384 US veterans with COVID‐19, estimating the burden of PASC to around 4.5% in nonhospitalized individuals, 22% in hospitalized individuals, and 36% in ICU‐treated individuals [19]. Studies assessing more specific outcomes after COVID‐19—including problems related to mental health, cardiovascular events, and general healthcare use—have observed an increased occurrence among individuals with more severe acute infections [20, 21, 22, 23]. In a Danish population‐based cohort study of 8983 SARS‐CoV‐2‐positive individuals not requiring hospital admission compared to SARS‐CoV‐2‐negative controls, positive individuals were not at an increased risk of initiating 12 out of 15 assessed medications, and no increased risk was observed for receiving 25 out of 27 assessed hospital diagnoses 2 weeks to 6 months after their SARS‐CoV‐2 test [24]. Our findings of a low occurrence of PCC diagnosis (1%) needing healthcare attendance among nonhospitalized individuals extend these findings. A similar healthcare attendance before and after the acute SARS‐CoV‐2 infection among individuals without a PCC diagnosis favors that we are not underestimating the occurrence of PCC in need of healthcare in this patient group.

Our finding of 32% of ICU‐treated individuals diagnosed with PCC indicates substantial sequelae after severe acute infection. In a recent Dutch study of health status 1 year after ICU admission in more than 200 patients, 74% reported physical symptoms, 26% mental symptoms, and 16% cognitive symptoms [25]. This study was based on patient‐reported outcome measures and self reports, whereas the main outcome in our study was a PCC diagnosis. As such, it is possible that a significant proportion of individuals not receiving a PCC diagnosis in our study experienced symptoms and sequelae after ICU treatment, indicated by our finding of increases in primary and outpatient specialist care also among ICU‐treated individuals not diagnosed with post COVID‐19. The occurrence of long‐term physical, cognitive, or mental health impairments persisting beyond intensive care hospitalization—known as post–intensive care syndrome (PICS)—has been reported to exceed 50% [26]. What possibly distinguishes PCC from PICS needs to be better understood [27]. This problem might be aggravated by the etiological focus of the diagnosis code, rather than focusing on the distinct clinical phenotypic manifestations [28, 29].

PCC has been found to be more prevalent among women [30, 31]. Reasons for this are largely unknown but have been suggested to include sex hormone differences [32]. In our study, PCC was less common among the elderly. Potential reasons might include difficulties in diagnosing the condition, and differences in healthcare‐seeking behavior and attitudes towards normal recovery and convalescence. The associations of previous outpatient healthcare usage and comorbidities and PCC could indicate differences in healthcare‐seeking behavior or diagnostic bias. Our finding of around 50%–60% of individuals with a PCC diagnosis having symptom diagnoses concordant with the WHO definition of PCC before the acute SARS‐CoV‐2 infection indicates that some of the disease burden could be wrongly attributed to COVID‐19 or that COVID‐19 accentuates pre‐existing conditions.

The increases in outpatient care usage 12 months after the acute infection in individuals with a PCC diagnosis indicate persistence of the condition well beyond the acute infection. These findings are in line with a report on 968 adult patients from a French cohort of individuals with verified infection, of whom 85% of individuals symptomatic 2 months after the infection still reported symptoms 1 year after symptom onset [33]. Similar findings have also been described in a study on patients discharged from the hospital with COVID‐19 across the UK, where only 29% of 807 participants assessed 1 year after discharge reported full recovery [34]. Further, an English cohort study of 456,002 individuals with COVID‐19 found general practitioner rates for sequelae after the acute infection to differ depending on the severity of the infection, with anxiety and depression persisting 9 months after the acute infection [23].

Strengths of our study include the population‐based design of individuals with verified SARS‐CoV‐2 infection across different severities of acute infection. Access to data on all positive SARS‐CoV‐2 tests in the study region and inpatient and outpatient care—including primary care—enabled robust classification of SARS‐CoV‐2 exposure status, comorbidities, and healthcare use. Access to primary care data is of particular importance because more than half of the patients received their PCC diagnosis in primary care exclusively.

Regarding limitations, it is unlikely that retrospective collection of diagnostic codes—even if they are collected from primary and specialist care, as in our study—adequately reflects the full spectrum of PCC. Yet, with the long follow‐up time, it is likely that we captured a relevant proportion of sequelae to SARS‐CoV‐2 through the usage of the PCC diagnosis. However, since a standardized clinical case definition of PCC was unavailable during large parts of the study period, the use of the PCC diagnosis may have varied between doctors and healthcare facilities, possibly resulting in diagnostic misclassification and/or underreporting of PCC. Also, symptom diagnoses might not be adequately registered by clinicians, or accurately capture symptoms consistent with PCC. Further, differences in follow‐up—with a more structured follow‐up in hospitalized and ICU‐treated individuals compared to nonhospitalized individuals—might affect the observed occurrence of PCC diagnosis. Taken together, the occurrence of PCC diagnosis observed herein likely underestimates the incidence of the condition and the proportion experiencing reduced quality of life following SARS‐CoV‐2 infection. Finally, we did not have information on the SARS‐CoV‐2 vaccination status available and as such could not evaluate the effect of vaccination on subsequent risk of post COVID‐19. Forty‐six percent of the study population was infected before the first vaccination was given in Sweden (27 December 2020), and by the end of inclusion, of new infections (31 July 2021), 48% of the Swedish population had received at least two vaccine doses [35].

In conclusion, the differences observed between nonhospitalized, hospitalized, and ICU‐treated individuals in how age, sex, comorbidities, and healthcare usage were associated with PCC diagnosis indicate heterogeneity in diagnostic procedures, etiology, clinical phenotypes, and trajectory of the disease. This should be accounted for in future updates of the case definition and diagnosis code of PCC. Further, the substantial differences in healthcare usage between individuals with and without a PCC diagnosis extending 1 year after the COVID‐19 infection motivate further studies with longer follow‐up to understand disease trajectories, resolution of symptoms, and healthcare needs following COVID‐19.

## Conflicts of interest

The authors declare that there is no conflict of interest that could be perceived as prejudicing the impartiality of the research reported.

## Author contributions

P. N. and P. H. had full access to all the data in the study and take responsibility for the integrity of the data and the accuracy of the data analysis. All authors have read and approved the manuscript. Study concept and design: all authors. Acquisition, analysis, or interpretation of data: all authors. Drafting of the manuscript: P. H., P. N., and F. G. Critical revision of the manuscript for important intellectual content: all authors. Statistical analysis: P. H. and F. G.

## Supporting information

Supporting Information

## Data Availability

The individual participant data underlying this article were subject to ethical approval and cannot be shared publicly. Data from the de‐identified administrative health registry are not freely available due to protection of the personal integrity of the participants.
