# Supplementary material for: Post COVID‐19 condition diagnosis: A population‐based cohort study of occurrence, associated factors, and healthcare use by severity of acute infection
Source: J Intern Med. 2022 Dec 7;293(2):246–58. doi: 10.1111/joim.13584 (PMC9877994; doi:10.1111/joim.13584)
Supplement: Supplementary file 1 — Supporting Information [file JOIM-293-246-s001.docx]

Supplementary material for

**Post COVID-19 condition diagnosis: A population-based cohort study of occurrence, associated factors, and healthcare use by severity of acute infection**

**Authors:** Pontus Hedberg, Fredrik Granath, Judith Bruchfeld, Johan Askling, Daniel Sjöholm, Michael Fored, Anna Färnert, Pontus Naucler

Correspondence to [pontus.hedberg@ki.se](mailto:pontus.hedberg@ki.se)

**Table of contents**

| **Content** | **Page** |
| --- | --- |
| eTable 1. Study definitions | 3-5 |
| **eFigure 1.** Associations between hospital/ICU length of stay (days) and post COVID-19 | 6 |
| **eFigure 2.** The occurrence of symptoms consistent with post COVID-19 before and after the acute infection in individuals with post COVID-19 | 7 |
| **eFigure 3.** ICD-10 chapters for the registered diagnoses during previous inpatient and outpatient healthcare use in individuals with and without post COVID-19 condition | 8 |
| **eFigure 4.** Most common diagnoses registered during previous inpatient and outpatient healthcare use in individuals with and without post COVID-19 condition | 9 |
| **eTable 2.** Characteristics of the matched cohorts stratified by severity of acute infection | 10-13 |
| **eFigure 5.** Standardized mean differences before and after matching in non-hospitalized individuals | 14 |
| **eFigure 6.** Standardized mean differences before and after matching in hospitalized individuals | 15 |
| **eFigure 7.** Standardized mean differences before and after matching in ICU-treated individuals | 16 |
| **eFigure 8.** Proportion of outpatient care related to post COVID-19 in individuals with post COVID-19 condition diagnosis | 17 |
| **Supplementary references** | 18 |

# eTable 1. Study definitions

| **Variable type** | **Variable** | **Data sources used** | **Missing data** | **Definition** | **Additional description** |
| --- | --- | --- | --- | --- | --- |
| Study population | Inclusion and exclusion criteria | VAL, SmiNet | No | - A first positive SARS-CoV-2 test from 1 March 2020 to 31 July 2021.  - 18 years or older when first tested SARS-CoV-2 positive. - Not moving in to or out from the Stockholm Region from 1,110 days before to 90 days after first positive SARS-CoV-2 test. - Alive 90 days after first positive SARS-CoV-2 test. - A follow-up of 90 days or more after discharge from a COVID-19 hospitalization. |  |
| Study outcome | Post COVID-19 condition | VAL | No | A post COVID-19 condition diagnosis (U09.9) registered any time from 90 to 360 days after first positive SARS-CoV-2 test, given by any healthcare professional in primary care, outpatient specialist care or inpatient care. |  |
| Severity of COVID-19 | Not hospitalized | SmiNet, VAL, SIR | No | No COVID-19 hospitalization according to the definition below. |  |
|  | Hospitalized |  |  | A U07.1/U07.2 code as main hospital discharge diagnosis, with a first positive SARS-CoV-2 test from 14 days before admission to the day of discharge. No treatment in the ICU during the hospitalization. |  |
|  | ICU-treated |  |  | A U07.1/U07.2 code as main hospital discharge diagnosis, with a first positive SARS-CoV-2 test from 14 days before admission to the day of discharge. Treated in the ICU during the hospitalization. |  |
| Post COVID-19 related symptoms | Abdominal pain | VAL | No | ICD-10: R10 | Both inpatient and outpatient ICD-10 codes considered.  Assessment window 300 to 30 days before the infection and 90 to 360 days after the infection. New-onset symptom was defined as any of the codes registered after the acute infection, without any of the codes registered before the infection. Codes with a higher resolution (i.e. G47.0 and G47.1 for G47) were included for all the listed ICD-10 codes. |
|  | Altered smell/taste |  |  | ICD-10: R43 |  |
|  | Anxiety |  |  | ICD-10: F40-F48 |  |
|  | Chest pain |  |  | ICD-10: R07.1, R07.2, R07.3, R07.4 |  |
|  | Cough |  |  | ICD-10: R05 |  |
|  | Depression |  |  | ICD-10: F30-F39 |  |
|  | Dizziness |  |  | ICD-10: R42 |  |
|  | Dyspnea |  |  | ICD-10: R06.0 |  |
|  | Fatigue |  |  | ICD-10: G93.3, R53 |  |
|  | Fever |  |  | ICD-10: R509 |  |
|  | Gastrointestinal issues (diarrhea, constipation, acid reflux) |  |  | ICD-10: K51, K58, K59.0, K59.1, K59.2, R11, R12, R19.4 |  |
|  | Headache |  |  | ICD-10: G43, G44, R51 |  |
|  | Joint pain |  |  | ICD-10: M25.5 |  |
|  | Myalgia |  |  | ICD-10: M79.1, M79.2, M79.6, M79.9, M79.9, R25.2 |  |
|  | Neuralgia |  |  | ICD-10: G50.0, M79.2, R52.2 |  |
|  | Paresthesia |  |  | ICD-10: R20.2 |  |
|  | Sleep disorder |  |  | ICD-10: G47 |  |
|  | Tachycardia/palpitations |  |  | ICD-10: I47, I49.5, R00.0, R00.2 |  |
|  | Tinnitus and other hearing issues |  |  | ICD-10: H93.1, H93.2 |  |
| Sociodemographic data | Region of birth | Statistics Sweden | Yes, for 0.33% of study subjects | According to the United Nations geoscheme |  |
|  | Residential area deprivation |  | Yes, for 0.40% of study subjects | According to the five area types defined by Statistics Sweden and Delegationen för segregation (Delmos).^1^ |  |
|  | Sick days during 2019 |  | Yes, for 0.36% of study subjects | Number of net days with sickness benefit from the Swedish Social Insurance Agency during year 2019. Sickness benefit is paid from 15 days after start of illness and onwards, whereas the first 14 days are paid by the employer. |  |
| Comorbidities | Asthma | VAL | No | ICD-10: J45 | Both inpatient and outpatient ICD-10 codes considered.  Assessment window 1,110 to 30 days before the first positive SARS-CoV-2 test for all ICD-10 codes and 390 to 30 days for ATC codes for immunocompromised state. Codes with a higher resolution (i.e. N18.1 and N18.2 for N18) were included for all the listed ICD-10 codes. |
|  | Cancer |  | No | ICD-10: C00-C26, C30-C34, C37-C39, C40-C80, C81-C96 |  |
|  | Cerebrovascular disease |  | No | ICD-10: I60-I69 |  |
|  | Chronic kidney disease |  | No | ICD-10: N18 |  |
|  | Chronic liver diseases |  | No | ICD-10: B180E, B180G, B181E, B181G, B182E, B182G, B188E, B188G, B189E, B189G, K70, K71.7, K74, K75.4, K760 |  |
|  | Chronic lung diseases |  | No | ICD-10: I26, I27.0, I27.2, J44, J47, J70.2, J70.3, J70.4, J84, J98.2, J99.0, M05.1 |  |
|  | Diabetes mellitus (type 1 or 2) |  | No | ICD-10: E10-E14 |  |
|  | Heart disease |  | No | ICD-10: I05-I08, I20-I22, I24-I28, I34-I37, I42, I44-I50 |  |
|  | Hypertension |  | No | ICD-10: I10-I15 |  |
|  | Immunocompromised state |  | No | ICD-10: D70-D72, D73.0, D80-D84, Z51.0, Z51.1   OR  ATC: Two or more prescriptions of H02- or L04-drugs 390 to 30 days before SARS-CoV-2 positive test and one or more prescriptions 120 to 30 days before SARS-CoV-2 positive test. |  |
|  | Mental health disorders |  | No | ICD-10: F20-F29, F30-F39, F40-F48 |  |
|  | Neurologic conditions |  | No | ICD-10: F01-F03, G10-G14, G20-G26, G30-G32, G70-G73, G80-G83. |  |
| Healthcare use | Inpatient care | VAL | No | All inpatient care provided in the region. | Assessment window 300 to 30 days before the infection and 90 to 360 days after the infection. For outpatient care, only visits to medical doctors were included. |
|  | Outpatient specialist care |  | No | All outpatient specialist care provided in the region. |  |
|  | Primary care |  | No | Around 94% of all outpatient primary care provided in the region. |  |

**Abbreviations:** ATC=Anatomical therapeutic chemical; ICD-10=International classification of diseases, tenth revision; SARS-CoV-2=Severe acute respiratory syndrome coronavirus 2

# eFigure 1. Associations between hospital/ICU length of stay (days) and post COVID-19

**Note:** For hospitalized individuals, the number of days represent the number of hospitalization days, whereas this for ICU-treated represents the number of days in the ICU. Cox proportional hazards regression models stratified on month of first positive SARS-CoV-2 test, adjusted for age (restricted cubic splines with 4 knots), sex, and the interaction between age and sex were used.

**Abbreviations:** ICU=intensive care unit; Ref=Reference

# eFigure 2. The occurrence of symptoms consistent with post COVID-19 before and after the acute infection in individuals with post COVID-19

# Abbreviations: ICU=Intensive care unit

**Note:** Previous symptoms were based on symptom diagnosis codes registered from 300 to 30 days before first positive SARS-CoV-2 test. New-onset symptoms were based on symptom diagnosis codes registered from 90 to 360 days after first positive SARS-CoV-2 test and not present 300 to 30 days before first positive SARS-CoV-2 test. The proportion represents the proportion of all individuals within that specific stratum that had such a diagnosis code registered. For each specific symptom, individuals with a previous history of the symptom were excluded from the denominator.

# eFigure 3. ICD-10 chapters for the registered diagnoses during previous inpatient and outpatient healthcare use in individuals with and without post COVID-19 condition

# Abbreviations: ICU=Intensive care unit

**Note:** All diagnoses registered during inpatient and outpatient care from 300 to 30 days before first positive SARS-CoV-2 test. The proportion represents the proportion of all individuals within that specific patient group that had such a diagnosis code registered.

# eFigure 4. Most common diagnoses registered during previous inpatient and outpatient healthcare use in individuals with and without post COVID-19 condition

# Abbreviations: ICU=Intensive care unit

**Note:** All diagnoses registered during inpatient and outpatient care from 300 to 30 days before first positive SARS-CoV-2 test. For each patient group (stratified by severity and post COVID-19 diagnosis or not), the 15 most common diagnosis codes were selected. These diagnosis codes were then characterized for all patient groups. The proportion represents the proportion of all individuals within that specific patient group that had such a diagnosis code registered.

**eTable 2. Characteristics of the matched cohorts stratified by severity of acute infection.**

|  | **Non-hospitalized (n=7852)** | | **Hospitalized  (n=2758)** | | **ICU-treated (n=720)** | |
| --- | --- | --- | --- | --- | --- | --- |
| **Variable** | **Post COVID-19  (n=1963)** | **Matched controls (n=5889)** | **Post COVID-19 (n=690)** | **Matched controls (n=2068)** | **Post COVID-19 (n=360)** | **Matched controls (n=360)** |
| Female sex | 1399 (71·3) | 4197 (71·3) | 322 (46·7) | 966 (46·7) | 111 (30·8) | 111 (30·8) |
| Age, years | 48·0 [40·0, 56·0] | 48·0 [40·0, 56·0] | 60·0 [52·0, 70·0] | 60·0 [52·0, 70·0] | 61·0 [53·0, 67·0] | 61·0 [53·0, 68·0] |
| 18-29 | 119 (6·1) | 357 (6·1) | 6 (0·9) | 18 (0·9) | 2 (0·6) | 2 (0·6) |
| 30-39 | 354 (18·0) | 1062 (18·0) | 25 (3·6) | 74 (3·6) | 11 (3·1) | 11 (3·1) |
| 40-49 | 591 (30·1) | 1773 (30·1) | 103 (14·9) | 309 (14·9) | 50 (13·9) | 50 (13·9) |
| 50-59 | 561 (28·6) | 1683 (28·6) | 192 (27·8) | 576 (27·9) | 110 (30·6) | 110 (30·6) |
| 60-69 | 257 (13·1) | 771 (13·1) | 186 (27·0) | 558 (27·0) | 113 (31·4) | 113 (31·4) |
| 70-79 | 64 (3·3) | 192 (3·3) | 116 (16·8) | 347 (16·8) | 70 (19·4) | 70 (19·4) |
| >80 | 17 (0·9) | 51 (0·9) | 62 (9·0) | 186 (9·0) | 4 (1·1) | 4 (1·1) |
| **Comorbidities** |  |  |  |  |  |  |
| Asthma | 230 (11·7) | 647 (11·0) | 118 (17·1) | 323 (15·6) | 51 (14·2) | 33 (9·2) |
| Cancer | 56 (2·9) | 153 (2·6) | 70 (10·1) | 217 (10·5) | 27 (7·5) | 32 (8·9) |
| Cerebrovascular disease | 22 (1·1) | 55 (0·9) | 26 (3·8) | 83 (4·0) | 12 (3·3) | 15 (4·2) |
| Chronic kidney disease | 14 (0·7) | 32 (0·5) | 39 (5·7) | 114 (5·5) | 10 (2·8) | 14 (3·9) |
| Chronic liver disease | 13 (0·7) | 34 (0·6) | 10 (1·4) | 29 (1·4) | 6 (1·7) | 5 (1·4) |
| Chronic lung disease | 33 (1·7) | 93 (1·6) | 53 (7·7) | 147 (7·1) | 20 (5·6) | 22 (6·1) |
| Diabetes (type 1 or type 2) | 92 (4·7) | 241 (4·1) | 130 (18·8) | 399 (19·3) | 89 (24·7) | 91 (25·3) |
| Heart disease | 95 (4·8) | 261 (4·4) | 109 (15·8) | 330 (16·0) | 57 (15·8) | 51 (14·2) |
| Hypertension | 298 (15·2) | 859 (14·6) | 276 (40·0) | 795 (38·4) | 146 (40·6) | 155 (43·1) |
| Immunocompromised state | 84 (4·3) | 234 (4·0) | 58 (8·4) | 190 (9·2) | 33 (9·2) | 27 (7·5) |
| Mental health disorder | 720 (36·7) | 2146 (36·4) | 193 (28·0) | 564 (27·3) | 80 (22·2) | 68 (18·9) |
| Neurological disease | 36 (1·8) | 100 (1·7) | 42 (6·1) | 138 (6·7) | 14 (3·9) | 12 (3·3) |
| Number of comorbidities |  |  |  |  |  |  |
| 0 | 875 (44·6) | 2658 (45·1) | 191 (27·7) | 612 (29·6) | 107 (29·7) | 118 (32·8) |
| 1 | 696 (35·5) | 2144 (36·4) | 175 (25·4) | 500 (24·2) | 94 (26·1) | 89 (24·7) |
| 2 | 258 (13·1) | 730 (12·4) | 154 (22·3) | 424 (20·5) | 70 (19·4) | 78 (21·7) |
| >2 | 134 (6·8) | 357 (6·1) | 170 (24·6) | 532 (25·7) | 89 (24·7) | 75 (20·8) |
| **Previous healthcare use** |  |  |  |  |  |  |
| Primary care |  |  |  |  |  |  |
| 0 visits | 624 (31·8) | 1793 (30·4) | 204 (29·6) | 658 (31·8) | 127 (35·3) | 131 (36·4) |
| 1-2 visits | 693 (35·3) | 2169 (36·8) | 256 (37·1) | 759 (36·7) | 133 (36·9) | 132 (36·7) |
| 3-4 visits | 328 (16·7) | 1029 (17·5) | 111 (16·1) | 332 (16·1) | 57 (15·8) | 54 (15·0) |
| >4 visits | 318 (16·2) | 898 (15·2) | 119 (17·2) | 319 (15·4) | 43 (11·9) | 43 (11·9) |
| Outpatient specialist care |  |  |  |  |  |  |
| 0 visits | 880 (44·8) | 2758 (46·8) | 249 (36·1) | 773 (37·4) | 150 (41·7) | 168 (46·7) |
| 1-2 visits | 555 (28·3) | 1646 (28·0) | 212 (30·7) | 641 (31·0) | 96 (26·7) | 95 (26·4) |
| 3-4 visits | 237 (12·1) | 632 (10·7) | 85 (12·3) | 238 (11·5) | 42 (11·7) | 41 (11·4) |
| >4 visits | 291 (14·8) | 853 (14·5) | 144 (20·9) | 416 (20·1) | 72 (20·0) | 56 (15·6) |
| Any inpatient visit | 94 (4·8) | 263 (4·5) | 80 (11·6) | 238 (11·5) | 37 (10·3) | 45 (12·5) |
| **Sociodemographic variables** |  |  |  |  |  |  |
| Days with sickness benefit during 2019 |  |  |  |  |  |  |
| 0 | 1563 (79·6) | 4718 (80·1) | 564 (81·7) | 1727 (83·5) | 301 (83·6) | 332 (92·2) |
| 1-30 | 178 (9·1) | 533 (9·1) | 55 (8·0) | 138 (6·7) | 14 (3·9) | 8 (2·2) |
| >30 | 222 (11·3) | 638 (10·8) | 71 (10·3) | 203 (9·8) | 45 (12·5) | 20 (5·6) |
| Region of birth |  |  |  |  |  |  |
| Africa | 37 (1·9) | 108 (1·8) | 30 (4·3) | 96 (4·6) | 20 (5·6) | 24 (6·7) |
| America | 75 (3·8) | 195 (3·3) | 34 (4·9) | 92 (4·4) | 24 (6·7) | 22 (6·1) |
| Asia/Oceania | 270 (13·8) | 818 (13·9) | 121 (17·5) | 377 (18·2) | 78 (21·7) | 74 (20·6) |
| Europe | 202 (10·3) | 594 (10·1) | 97 (14·1) | 283 (13·7) | 56 (15·6) | 61 (16·9) |
| Sweden | 1379 (70·2) | 4174 (70·9) | 408 (59·1) | 1220 (59·0) | 182 (50·6) | 179 (49·7) |
| Residential area type |  |  |  |  |  |  |
| 1 (most deprived) | 28 (1·4) | 73 (1·2) | 22 (3·2) | 67 (3·2) | 9 (2·5) | 10 (2·8) |
| 2 | 126 (6·4) | 319 (5·4) | 79 (11·4) | 240 (11·6) | 57 (15·8) | 60 (16·7) |
| 3 | 252 (12·8) | 738 (12·5) | 112 (16·2) | 309 (14·9) | 67 (18·6) | 66 (18·3) |
| 4 | 1138 (58·0) | 3471 (58·9) | 342 (49·6) | 1034 (50·0) | 166 (46·1) | 178 (49·4) |
| 5 (least deprived) | 419 (21·3) | 1288 (21·9) | 135 (19·6) | 418 (20·2) | 61 (16·9) | 46 (12·8) |

**Note:** Numeric values are presented as median (interquartile range), and categorical values are presented as number (percentage).

**Abbreviations:** COVID-19=Coronavirus disease 2019; ICU=Intensive care unit

# eFigure 5. Standardized mean differences before and after matching in non-hospitalized individuals.

#

**Note:** Exact matching was used for month of positive test, age group and sex. These variables are not shown in the figure. The vertical dashed line represents a standardized mean difference of 0.05 and the vertical solid line represents a standardized mean difference of 0.10. **eFigure 6. Standardized mean differences before and after matching in hospitalized individuals.**

#

# Note: Exact matching was used for month of positive test, age group and sex. These variables are not shown in the figure. The vertical dashed line represents a standardized mean difference of 0.05 and the vertical solid line represents a standardized mean difference of 0.10. eFigure 7. Standardized mean differences before and after matching in ICU-treated individuals.

#

**Note:** Exact matching was used for month of positive test, age group and sex. These variables are not shown in the figure. The vertical dashed line represents a standardized mean difference of 0.05 and the vertical solid line represents a standardized mean difference of 0.10.

**eFigure 8. Proportion of outpatient care related to post COVID-19 in individuals with post COVID-19 condition diagnosis**

**Note:** The proportion of all primary care and outpatient specialist care visits with a post COVID-19 condition diagnosis or a symptom diagnosis consistent with post COVID-19 (see eTable 1 for diagnosis codes used).

**Abbreviations:** ICU= Intensive care unit**References**

1 Delmos. Så mäter och följer du segregation Användarhandbok för Segregationsbarometern. 2021.
